# Polymorphisms Involved in Insulin Resistance and Metabolic Inflammation: Influence of Nutrients and Dietary Interventions

**DOI:** 10.3390/metabo15040245

**Published:** 2025-04-02

**Authors:** Graziela Biude Silva Duarte, Gabriela de Freitas Laiber Pascoal, Marcelo Macedo Rogero

**Affiliations:** Department of Nutrition, School of Public Health, University of São Paulo, São Paulo 01246-904, Brazil; gbiude@usp.br (G.B.S.D.); laiber@usp.br (G.d.F.L.P.)

**Keywords:** nutrition, polymorphism, metabolic inflammation

## Abstract

Insulin resistance (IR) is a metabolic disorder characterized by an impaired response to insulin. This condition is associated with excess adiposity and metabolic inflammation, contributing to an increased risk for related chronic diseases. Single-nucleotide polymorphisms (SNPs) can affect genes related to metabolic pathways which are related to IR and the individual response to nutrients and dietary patterns, affecting metabolic inflammation and insulin sensitivity. This narrative review explores the current evidence on interactions between genetic variants and dietary factors, specifically their effects in modulating IR and metabolic inflammation. A comprehensive search of the literature was conducted in PubMed, Google Scholar, and Web of Science, and a total of 95 articles were reviewed. The key findings reveal that SNPs in the *TCF7L2*, *ADIPOQ*, and *TNF* genes significantly influence metabolic responses and modulate the effects of the Mediterranean diet on biomarkers of inflammation and IR. Genotype-dependent variations in IR and inflammation biomarkers were observed in the response to different diets for SNPs in the *TCF7L2*, *ADIPOQ*, and *TNF* genes. Additionally, polygenic risk scores (PRSs) can also predict the response to the intake of nutrients and specific diets, and offer a promising tool for assessing genetic predisposition to IR. This review underscores the pivotal role of an individual’s genetic background in the effects of their nutrient intake and in the responses to dietetic interventions, thereby laying the foundation for personalized and effective nutritional strategies tailored to each individual’s necessity in mitigating IR and its associated risk factors for chronic diseases.

## 1. Introduction

Insulin resistance (IR) is a complex metabolic disorder characterized by the body’s inadequate response to insulin, a crucial peptide hormone responsible for maintaining glucose homeostasis. This condition is marked by an impaired biological response to insulin in target tissues, notably the liver, skeletal muscle, and white adipose tissue [1]. Consequently, individuals with IR often exhibit hyperglycemia and hyperinsulinemia as a result of impaired glucose uptake. IR affects approximately 25–35% of the Western population and is associated with excess adiposity and an increased risk of obesity-related diseases, such as type 2 diabetes (T2D), cardiovascular disease, and certain types of cancer [2,3].

Obesity, particularly visceral adipose tissue accumulation, is one of the major risk factors for IR development. This excess adiposity triggers a systemic low-grade chronic inflammatory response [4] that is mediated by pro-inflammatory cytokines, such as IL-1β, IL-6, and TNF-α, which are secreted by both recruited and resident macrophages in the white adipose tissue, liver, and skeletal muscle [5]. This complex interplay of weight gain and chronic low-grade inflammation triggers a condition known as metabolic inflammation, which highlights the systemic nature of this condition and its broad impacts on health [6]. The increased levels of these cytokines impair the biological response to insulin stimulation through mechanisms such as IRS-1 degradation, serine/threonine protein kinase activation, decreased PPAR-γ expression, and impaired GLUT4 translocation [7].

Advances in genetic studies over recent decades have allowed for the identification of several candidate genes involved in metabolic pathways that are related to energy balance, glucose homeostasis, and inflammatory response. The discovery of single-nucleotide polymorphisms (SNPs), which represent approximately 90% of all known sequence variations in the human genome, has been particularly significant. These variations in a single position of the DNA sequence, occurring in at least 1% of the population, have been associated with an increased risk of developing chronic metabolic diseases and their complications. The understanding of how SNPs are linked to these conditions has allowed researchers to better understand individuals’ susceptibility to metabolic disorders such as IR [8].

Genome-wide association studies (GWASs) have significantly advanced our understanding of T2D, identifying approximately 88 loci associated with the risk of this disease. These loci are involved in insulin secretion and β-cell function, as well as in insulin signaling and action in other cell types, such as hepatocytes, adipocytes, myocytes, and neurons, highlighting the multifactorial nature of the pathophysiology of T2D [9,10].

Among them, SNPs on the transcription factor 7-like 2 (*TCF7L2*) exhibit the most robust association with the risk of developing T2D, affecting both the β-cell function and secretion [11]. In this scenario, carriers of the C allele for the rs2943641 SNP, which is adjacent to the insulin receptor substrate 1 (*IRS1*) gene, have been associated with IR with hyperinsulinemia in Europeans [8].

Further exploration in a large-scale study involving 76,000 participants identified 16 distinct genetic loci associated with fasting glucose regulation and two loci specifically linked to fasting insulin concentration. Of particular significance were the loci near the glucokinase regulatory protein (GCKR), a key regulator of hepatic glucose metabolism, and a novel locus near insulin-like growth factor-1 (IGF1), which were identified as contributors to IR. This discovery was corroborated across 14 cohorts involving 29,000 non-diabetic individuals. A subsequent study categorized the risk loci into distinct clusters, which contained the peroxisome proliferator-activated receptor gamma (*PPARG*), transcription factor *KLF14*, *IRS1*, and *GCKR* genes, being particularly notable for its strong association with IR [8,12].

Therefore, several other SNPs have been demonstrated to play crucial roles in the pathogenesis and progression of IR [13,14]. Among these genetic variants, SNPs associated with abdominal obesity, inflammation, and the lipid profile, identified in GWAS studies, have emerged as promising candidates for understanding the genetic architecture of metabolic disorders [15,16]. This includes SNPs in genes such as the fat mass and obesity-associated (*FTO*) (rs9939609), melanocortin 4 receptor (*MC4R*) (rs17782313), tumor necrosis factor-alpha (*TNF)* (rs1800629), and *IL6* (rs1800795) [17,18,19]. These interconnected studies demonstrate the intricate complexity of the genetic factors that contribute to IR, emphasizing the dynamic and multifaceted interactions between various genes and their collective impact on the disease’s pathophysiology [8].

Recent studies have highlighted the pivotal role of dietary patterns and specific nutrient intake in modulating the expression of genes linked to IR and metabolic inflammation [20,21,22]. Dietary components, particularly long-chain polyunsaturated fatty acids (PUFAs), bioactive compounds, and both soluble and insoluble dietary fiber, can significantly influence the expression of key genes involved in inflammation and insulin signaling pathways [11,22,23,24]. Omega-3 fatty acids, particularly eicosapentaenoic acid (EPA) and docosahexaenoic acid (DHA), have the capacity to downregulate the expression of pro-inflammatory cytokines, potentially mitigating the effects of pro-inflammatory genetic variants like TNF-α and IL-6 [25,26]. Concurrently, diets rich in refined carbohydrates can exacerbate hyperinsulinemia by influencing the expression of genes such as *IRS1* and *GCKR*, further complicating the metabolic landscape in individuals who are predisposed to IR [27,28,29].

In this context, to further elucidate the nuanced interplay between diet, nutrients, and genetic polymorphisms influencing IR and metabolic inflammation, an investigation of key genes with well-established roles in relevant pathways is relevant. The *TCF7L2* and *IRS* genes, for example, are directly involved in glucose regulation, while the *IL6* and *TNF* genes are associated with the inflammatory response and have an important association with IR and metabolic dysfunction. Thus, this narrative review aims to comprehensively summarize the polymorphisms involved in IR and metabolic inflammation, as well as to understand the influence of nutrients and dietary patterns on these biological processes.

## 2. Materials and Methods

**Figure 1:**
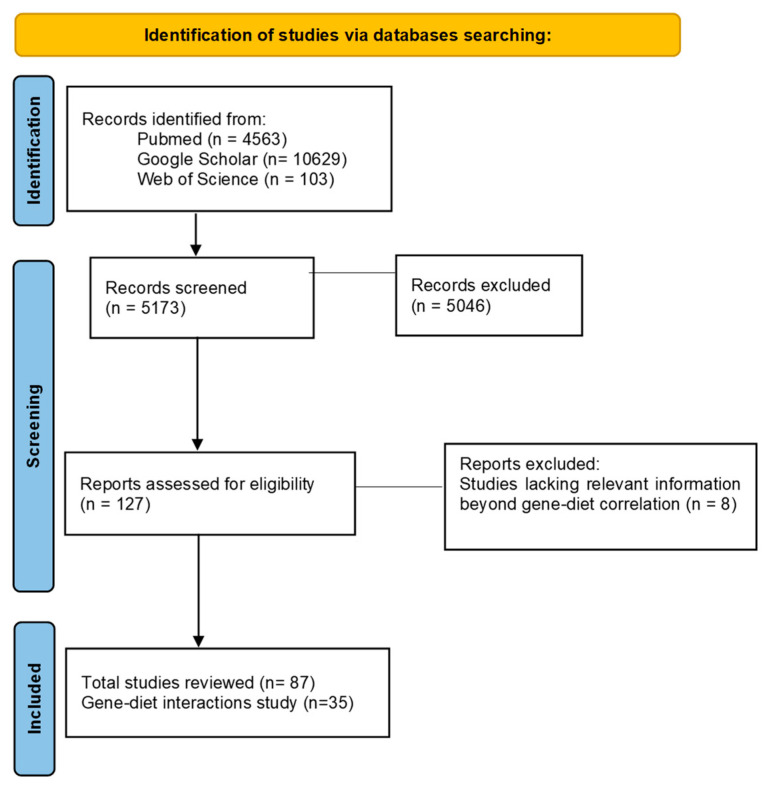
PRISMA flowchart of the study selection process.

This narrative review was conducted using a comprehensive search strategy across multiple databases including PubMed, Google Scholar, and Web of Science. The search terms used included “insulin resistance”, “metabolic inflammation”, “GWAS AND insulin resistance”, “SNPs AND insulin resistance”, “SNPs AND insulin resistance AND metabolic inflammation”, “nutrigenetics AND insulin resistance”, “SNP AND diet AND insulin resistance”, “SNP AND dietary pattern AND insulin resistance”, “nutrient-gene AND insulin resistance”, and “polygenic risk score AND diet”. A total of 95 eligible studies published between 2000 and 2025 were reviewed. Of these, 34 are meta-analyses, systematic reviews involving human subjects, and clinical trials that investigated the influence of specific nutrient intakes or dietary interventions (including specific nutrients, foods, or diets) on SNPs in genes related to IR and metabolic inflammation. No language restrictions were applied. We excluded studies conducted in animals or in vitro, that were not peer-reviewed, that were conference abstracts, or for which the articles were not available in full text (Figure 1).

## 3. Results and Discussion

### 3.1. IR and Metabolic Inflammation

Under normal physiological conditions, insulin is secreted by pancreatic β-cells in response to increased concentrations of glucose and other metabolites in the blood to regulate glucose uptake, promote glycogen synthesis, inhibit hepatic glucose production, and modulate lipid and protein metabolism, thereby maintaining systemic homeostasis [30,31]. The insulin signaling pathway begins with its binding to the insulin receptor tyrosine kinase on the plasma membrane of target cells, which, after undergoing autophosphorylation, promotes the recruitment of specific proteins such as IRS-1 and protein tyrosine phosphatase 1B (PTP1B). The induction of IRS-1 phosphorylation occurs through different insulin-activated kinases, such as protein kinase C (PKC) and protein kinase B (PKB or Akt), among others, whose intracellular events culminate in the regulation of glucose uptake through the translocation of glucose transporter 4 (GLUT4) from the cytoplasm to the plasma membrane [32,33].

Skeletal muscles, white adipose tissue, and the liver, which are directly influenced by insulin, play distinct roles in maintaining metabolic and glycemic homeostasis [30,34]. In individuals with normal insulin sensitivity, the signaling pathways of this hormone are activated, playing a crucial role in the regulation of glucose production in the liver. Additionally, these pathways trigger the uptake of glucose into fat and muscle cells, and thereby maintain glucose homeostasis [35].

Alterations or failures in the insulin signaling pathway lead to a reduced sensitivity to insulin in insulin-dependent tissues, particularly skeletal muscle, white adipose tissue, and the liver. This diminished responsiveness impairs the efficient uptake and utilization of glucose, culminating in a pathophysiological state that is characterized by chronic hyperglycemia and compensatory hyperinsulinemia. This metabolic disorder, known as IR, is the primary cause of T2D, and other chronic diseases can also contribute to the development and progression of this condition [32,36]. The methods for diagnosing IR include the hyperinsulinemic-euglycemic clamp (HEC) method as the gold standard, but due to its complexity of application, it is not common in clinical practice. However, other indexes are feasible and widely used for epidemiological studies and in clinical practice such as the homeostasis model assessment of insulin resistance (HOMA-IR), the quantitative insulin sensitivity check index (QUICK), and the triglyceride-glucose index (TyG) [37].

Abdominal obesity plays a significant role in the development of IR due to the excessive accumulation of adipose tissue. Individuals with abdominal obesity have an increased lipolytic activity of adipose tissue which results in elevated plasma free fatty acids concentrations (FFAs) and intracellular lipid accumulation. The rise of FFAs can activate protein kinases, for example, the c-Jun N-terminal kinase (JNK), which can induce IR due to the serine and threonine phosphorylation of insulin receptor substrate (IRS)-1 (e.g., Ser-307) and IRS-2 (e.g., Thr-348, Ser-488) in rodents and of IRS-1 (e.g., Ser-312) in humans, which downregulates the insulin-stimulated tyrosine phosphorylation of IRS proteins. Consequently, the inhibition of insulin-driven signal transduction occurs and then impairs the entry of glucose into the cell, resulting in its accumulation in the blood [38,39]. Evidence shows that a 10% reduction in the body mass index (BMI) improves IR in patients with obesity and with a diagnosis of T2D [40].

White adipose tissue (WAT) functions as an endocrine organ that actively secretes a diverse array of cytokines (adipokines) with profound pro-inflammatory actions, such as TNF-α, IL-1β, and IL-6, and s reduced levels of those with anti-inflammatory effects, such as IL-10 and adiponectin. In obesity, this inflammatory process is characterized by a chronic, systemic, and low-grade reaction caused by the excess of determined nutrients, such as saturated fatty acids (SAFAs), and the accumulation of metabolic byproducts. These factors orchestrate the activation of complex inflammatory signaling cascades, notably the nuclear factor kappa B (NF-κB) and JNK pathways, ultimately contributing to metabolic inflammation [41,42].

The increase and maintenance of this chronic low-grade inflammation is important in the etiology of IR in obesity. Toll-like receptor (TLR)-4 contributes to IR by increasing the gene expression of pro-inflammatory cytokines, such as TNF-α, IL-1β, IL-6, IL-8, and IL-12, which, in turn, work as endogenous inflammatory mediators by interacting with receptors found in different target cells. Further, IL-6 also contributes to IR in this context by inducing cytokine signaling suppressor protein 3 (SOCS3), which interacts with tyrosine-phosphorylated proteins as an insulin receptor and reduces the phosphorylation of IRS-1 tyrosine. In consequence, this reduction weakens the connection between IRS-1 and the insulin receptor, as well as with the phosphatidylinositol-3-kinase (PI3K) [38,42,43,44].

IL-1β is a pro-inflammatory cytokine that is produced mainly by monocytes and macrophages and is also involved in IR. In conditions of hyperglycemia, the concentrations of IL-1β are increased. In this context, it is known that IL-1β binds to interleukin-1 receptor type (IL-1R1), which activates JAK proteins kinases, and thus stimulates the activation and translocation of NF-κB to the nucleus, increasing the gene expression of pro-inflammatory mediators. In addition, this cytokine impairs the insulin signaling in peripheral tissues, leading to reduced insulin sensitivity and β-cell secretion. On the other hand, PPAR-γ, which participates in the expression of inflammatory response and glucose metabolism genes, has an opposite effect on IR by suppressing the transcription activity of NF-κB and attenuating the inflammatory response by interacting with NF-κB p65 to induce ubiquitination and degradation. Additionally, PPAR-γ can upregulate the IRS proteins and improve IR induced by obesity [4].

### 3.2. Gene–Nutrient Interactions in IR: Evidence of Functional Consequences of Genetic Polymorphism

#### 3.2.1. TCF7L2

TCF7L2 is a transcription factor that is considered to be one of the most important candidate genes related to the risk of T2D in GWAS studies [45]. In humans, the *TCF7L2* gene is in chromosome 10q25.3, has 18 exons, and presents a highly conserved sequence region that corresponds to functional domains. *TCF7L2* is expressed in various tissues, such as the islets of Langerhans and the liver, and plays a role in the Wnt signaling pathway, regulating several genes involved in incretin production and blood glucose homeostasis. In the context of IR, reduced mRNA levels of *TCF7L2* in the pancreatic islet are related to increased β-cell apoptosis and a decrease in the proliferation of β-cells that reduces the insulin secretion [46]. Compelling evidence demonstrates that the inhibition of TCF7L2 activity in human or animal cell lines reduces insulin secretion in response to glucose [47].

The SNP rs7903146 (C > T) is a genetic variant in which the T allele is significantly associated with the risk of T2D in Caucasians, East Asians, South Asians, and other ethnicities [48,49]. In a southern Brazilian population, the frequency of the T allele of the *TCF7L2* gene was significantly higher (*p* < 0.001) in patients with T2D compared to non-diabetic controls [50]. A meta-analysis also demonstrates that the T allele was associated with gestational diabetes mellitus (GDM) in white, Hispanic/Latino, and Asian populations [51].

In observational studies, the relationship between dietary factors, genetics, and the risk of type 2 diabetes (T2D) has been extensively explored. A study conducted with an Algerian population investigated the relation between rs7903146, diet, and T2D risk. The T allele was associated with an increased risk of T2D, even after adjusting for BMI. The carriers of the T allele that had a greater consumption of desserts and milk presented an increased risk for T2D compared to those who had a lower consumption of these foods [52].

Another observational study, using data from the LIPGENE-SU.VI.MAX study, revealed that women carriers of the T allele for SNP rs7903146 in the *TCF7L2* gene demonstrated 66% more risk for developing metabolic syndrome and an increased concentration of fasting insulin, HOMA-IR, waist circumference, and blood pressure compared to CC genotype carriers. A high intake of SAFA was associated with more impaired insulin sensitivity in the T allele carriers, but this association was more significant in those with the lowest intake of SAFA [53]. In a study with EPIC-Potsdam participants genotyped for rs7903146, the daily intake of whole grains (50 g/portion) was associated with a 14% lower risk of developing T2D in the CC carriers, while the individuals with the T allele did not present this benefit association. These data suggest that the T allele may attenuate the protector effect of whole grains in T2D risk [54].

Conversely, randomized clinical trials provided evidence of direct dietary intervention effects on metabolic outcomes when stratified by genotype. A clinical trial involving 7.018 participants from the PREDIMED study explored the influence of adhesion to the Mediterranean diet (MedDiet) in relation to the SNP rs7903146. The authors verified that, when the adherence to MedDiet was low, the TT carriers presented higher fasting glucose concentrations compared to the CC+TT genotypes. However, this genotype-associated effect on fasting glucose was not observed in individuals with high adherence [55]. Another study investigated the relationship between adherence to the MedDiet and the SNPs rs7903146 and rs12255372 (G > T) in the *TCF7L2* gene. The results revealed that, among individuals with high adherence to the MedDiet, the presence of the T allele for both SNPs was associated with a significantly lower weight, BMI, and waist circumference [56]. Barabash et al. investigated whether rs7903146 modulates the effect of MedDiet adherence on GDM risk. The results showed that women carrying the T allele who had high adherence to the MedDiet early in pregnancy have a significantly lower risk of developing GMD compared to those with low adherence. This effect was not observed for CC genotype carriers [57].

In a randomized clinical trial, adults and elderly participants with T2D were stratified and randomly assigned to the dietary approach to stop hypertension (DASH) group or a legume-based DASH group for a 16-week period. All participants underwent genetic screening for the SNP rs7903146 in the *TCF7L2* gene. The plasma pro-inflammatory biomarkers TNF-α and IL-6 and the oxidative stress indicator malondialdehyde (MDA) were reduced after consuming the legume-based DASH diet in comparison to the DASH diet. Notably, these beneficial effects were observed regardless of *TCF7L2* genotype status, suggesting a robust dietary intervention effect independent of genetic predisposition [58].

#### 3.2.2. SLC30A8 (ZnT8)

Zinc plays an essential role in insulin secretion and action, as well as in the signaling pathways related to this condition [59]. Two zinc transporters located in the plasma membrane cell are involved in this regulation. ZIP14 (SCL39A14) is responsible for transporting plasma zinc into the cytoplasm of β-cells, while ZnT8 has the function of transporting zinc from the cytoplasm to the insulin secretory granules, which thereby promotes the formation of the hexameric Zn-insulin complex. Consequently, ZnT8 is considered an essential protein for the synthesis, storage, and action of insulin [60,61].

GWAS studies revealed that a genetic variant in the *SLC30A8* gene rs13266634 (C > T) in nucleotide 973 results in the encoding of a tryptophan instead of an arginine at codon 325 of the protein (20). The C allele has been strongly associated with an increased risk of T2D in several populations. Research indicates that individuals carrying this allele often present impaired insulin secretion, a reduction in β-cell function, and glucose intolerance. Evidence demonstrates that carriers of the C allele for the rs7903146 polymorphism in the *TCF7L2* gene exhibit a 17% increased risk of developing T2D per allele [62,63,64].

A prospective study of 26.132 individuals without a history of cardiometabolic disease found that zinc supplementation was associated with a reduced risk of T2D. This effect was particularly pronounced among individuals with the TT genotype for the rs13266634 SNPcompared to non-supplement users carrying the CC genotype [65]. In an intervention study, adults and elderly individuals with the CT/TT genotype for the SNP rs13266634 who received 50 mg of oral zinc acetate (2×/day) for 14 weeks showed a 15% (*p* = 0.04) increase in fasting insulin concentration. There was no significant difference in serum zinc concentrations between individuals with different genotypes [66]. Another study investigated the impact of the SNP rs13266634 on insulin concentration and lipids before and after a high-fat mixed macronutrient tolerant test (MMTT) in healthy adults and elderly individuals from California (United States of America). The authors observed that men with the CC genotype presented lower non-esterified fatty acid (NEFA) concentrations than men with the CT or TT genotype. However, the TT genotype was associated with a slower removal of triglycerides from the circulation in men after the MMTT, suggesting a negative impact on postprandial triglyceride metabolism [67].

A meta-analysis using cross-sectional data from 14 cohort studies identified a significant interaction between rs11558471 (A > G) in the *SLC30A8* gene and the total zinc intake on fasting glucose levels. Specifically, the results indicate that, in individuals carrying the A allele, each additional 1 mg of total zinc intake is associated with a modest reduction in the fasting blood sugar level (*p* = 0.005) [68].

While zinc plays a critical role in insulin signaling and secretion, the clinical evaluation of zinc biomarkers and possible interventions for IR considering the genetic background remains little explored. In the absence of a gold-standard biomarker for assessing zinc status, this comprehensive approach may help more accurately identify individuals at risk of zinc deficiency and potentially impact the prognosis of IR.

#### 3.2.3. IRS-1

Insulin receptor substrate 1 (IRS1) is a signaling adapter protein encoded by the IRS1 gene. It mediates signals from insulin and insulin-like growth factor-1 (IGF-1) receptors to downstream pathways, such as the phosphatidylinositol 3-kinase (PI3K)/protein kinase B (Akt) and extracellular signal-regulated kinases (Erk)-mitogen-activated protein kinase (MAPK) pathways, regulating essential cellular processes. IRS1 plays a crucial role in insulin signaling, and defects in IRS1 can disrupt this pathway, contributing significantly to insulin resistance, metabolic imbalance, and diabetic complications [69].

SNPs in the *IRS1* gene can significantly influence insulin sensitivity and, consequently, the risk of developing IR and T2D. A meta-analysis indicated a significant association between the SNP rs1801278 (G > A) and the recessive (AA versus GA + GG; *p* = 0.043) and codominant models (AA versus GG, *p* = 0.007) in Asiatic and Caucasian populations. In the same study, the rs2943641 (T > C) SNP exhibited a significant association with T2D in Caucasians only and in the codominant model (CT versus CC; *p* = 0.0023) [70]. Another meta-analysis indicated that the SNP rs1801278 is associated with the risk of DMG, but only in the recessive model (OR = 0.37; *p* = 0.030) [28].

A few studies investigated the effects of these SNPs and nutrients and diets in the context of IR. Ericson et al. evaluated the interaction between the SNP rs2943641 (T > A) and macronutrients in the incidence of T2D in a population of 15,227 adult women and 9614 adult men without prevalent diabetes in the south of Sweden with a follow-up of 12 years, with 1567 incident cases of T2DM identified. In women, the T allele was associated with a reduced risk only in the lower tertiles of carbohydrate intake (p-interaction = 0.01). In men, the T allele was associated with a reduced risk in the lowest tertile of fat intake (p-interaction = 0.02). These results showed that rs2943641 interacts with the carbohydrate and fat intake, affecting the incidence of T2D in a gender-specific manner [71].

A pilot study involving 358 Brazilian adults investigated the impact of the SNP rs2943634 (A > C) in the *IRS1* gene. The findings revealed that the A allele was associated with a reduced intake of carbohydrates following a dietary counseling program, suggesting that the SNP rs2943634 may influence the dietary response by affecting carbohydrate metabolism [72].

*IRS1* SNPs (rs2943641 and rs7578326 [A > G]) were evaluated in two populations with European and Puerto Rican ancestry. In the European ancestry population, the rs7578326 G-allele carriers, the rs2943641 T-allele carriers, and their haplotype G-T carriers had a significantly lower risk of IR and MetS only when the dietary saturated fatty acid–carbohydrate ratio was low (≤0.24). In both populations, the rs7578326 G-allele carriers had a lower risk of MetS than the non-carriers only when dietary monounsaturated fatty acids (MUFAa) were lower than the median intake of each population [27].

#### 3.2.4. TNF-α

SNPs at inflammatory biomarker genes have been associated with alterations in plasma levels, such as increased TNF-α and IL-6 concentrations, which contribute to chronic low-grade inflammation and metabolic dysfunction, ultimately influencing the risk of chronic diseases [73]. Some SNPs in the *TNF* gene, such as the rs1800629 (G > A), rs361525 (G > A), rs1799724 (C > T), and rs1799964 (T > C) allele carriers, are associated with higher *TNF-α* expression [74]. Evidence indicates that rs1800629 (G > A) can influence TNF-α expression levels, subsequently affecting inflammation and insulin signaling pathways [75,76]. The rs1800629 (G > A) polymorphism involves a substitution of guanine (G) to adenine (A), leading to a 2–3-fold increase in the transcriptional activity of TNF-α in response to bacterial lipopolysaccharide (LPS) stimulation.

The chronic consumption of the MedDiet interacts with the *TNF* rs1800629 SNP to influence plasma triglycerides (TGs), improving metabolism and inflammation biomarkers. Specifically, individuals with the GG genotype from Northern Germany who initially expressed higher TG and hsCRP plasma concentrations showed improvement in these biomarkers after 1 year of dietary intervention [77].

Dietary fatty acids (FAs) can modulate inflammation, depending on their chemical structure and classification. The *TNF* rs1800629 SNP interacts with stearic acid and total SFA. Oki and collaborators observed that carriers of the A allele of the rs1800629 polymorphism had a higher odds ratio (OR) of being in the inflammation group in two specific instances: (1) when plasma stearic acid concentrations were elevated and (2) when total plasma SAFA concentrations were increased [74]. These findings suggest an interaction between the TNF-α polymorphism and the plasma lipid composition in regulating inflammatory biomarkers. Stearic acid can be obtained from diet but is also synthesized endogenously through de novo lipogenesis (DNL). The high consumption of alcohol and carbohydrates, particularly fructose and simple carbohydrates, can trigger DNL and its products [78,79]. The circulating stearic acid was associated with BMI, waist circumference, TG, and HOMA-IR at baseline and with diabetes risk in prospective association [74]. Consequently, dietary interventions can play a crucial role in managing the inflammatory and metabolic consequences of *TNF-α* polymorphisms.

#### 3.2.5. TLR-4

The TLR family includes pathogen-specific receptors that are expressed in various tissues, such as the liver, adipose tissue, skeletal muscle, vasculature, pancreatic β cells, brain, macrophages, neutrophils, mast cells, B lymphocytes, and intestinal epithelium [12,16,80]. Toll-like receptor 4 (TLR4) is encoded by the *TLR4* gene located on chromosome 9 at 9q33.1 33. TLR4 functions as a pattern-recognition receptor that can be activated by LPS, which leads to the secretion of critical pro-inflammatory cytokines [80,81,82].

SNPs within the *TLR4* gene, particularly those located in the coding region, such as rs5030717 (A > G) and rs5030718 (C > T), play a significant role in modulating the receptor’s response to LPS [83]. This modulation can influence an individual’s susceptibility to inflammatory diseases and may provide predictive insights into the risk of complications, such as hypertension, nephropathy, and dyslipidemia, in T2D [84].

Genetic variation in *TLR4* may affect the relationship between dietary lipids and MetS, potentially influencing how IR develops in response to dietary factors. The dietary intake of healthy young men and women from various ethnocultural backgrounds was estimated using a semiquantitative food frequency questionnaire, and fasting blood samples were collected for genotyping and biomarker measurement. The intronic polymorphism rs5030728 altered the relationship between dietary SFAs and HDL-c. Specifically, SFA intake was inversely associated with HDL-c concentration among individuals who were homozygous for the G allele, whereas a positive relationship was observed for heterozygotes [85]. Few studies have examined the correlation between *TLR4* polymorphisms and diet, highlighting a need for further research in this area to better understand these interactions and their implications for IR and metabolic inflammation.

#### 3.2.6. IL-6

IL-6 is a pleiotropic cytokine that is mainly produced by T cells and macrophages, and is mapped to human chromosome 7p15–p21 [86]. IL-6 contributes to the development of IR and the pathogenesis of T2D via regulating inflammatory responses. Knockout experiments revealed elevated IL-6 expression in insulin-resistant individuals. Also, studies have shown that high levels of IL-6 were detected in the plasma of patients with T2D, and thus associated with its complication [86,87].

The IL-6 gene promoter polymorphism rs1800795 (G > C) represents a critical genetic variant that has been extensively studied across diverse populations for its association with T2D [87,88,89,90]. A recent study involving 36 pregnant women revealed that obese pregnant women share genetic similarities with T2D patients, suggesting an increased susceptibility to pregnancy-related complications. The GG genotype of the rs1800795 polymorphism proved to be the most frequent in all groups, except for women with normal pregnancy and BMI, who have the CC genotype at a 100% rate. This result suggests that the C allele may be protective, while the G allele, especially in its homozygous form, is linked to pregnancy issues [91].

The *IL6* SNP rs1800795 was genotyped in 242 women who were overweight/obese (BMI: 27–45 kg/m^2^) and underwent a one-year intervention with three diets: a lower fat (20% energy) and higher carbohydrate (65% energy; a lower carbohydrate (45% energy) and higher fat (35% energy); or a walnut-rich (18% energy from walnuts) with higher fat (35% energy) and lower carbohydrate (45% energy). The plasma IL-6 levels were measured at baseline, 6, and 12 months. At baseline, individuals with the CC genotype had lower IL-6 levels than those with the GC or GG genotypes (*p* < 0.03; 2.04 pg/mL vs. 2.72 pg/mL), but this difference was not significant after adjusting for BMI. No significant interaction between rs1800795 and the time or diet was observed. Thus, although rs1800795 is associated with IL-6 levels, the SNP did not modify the IL-6 response to diet or weight loss, reinforcing the effectiveness of these strategies in reducing IL-6 regardless of the genotype [92].

#### 3.2.7. PPAR-γ

Peroxisome proliferator-activated receptor gamma (PPAR-*γ*) represents a master regulator within the PPAR nuclear receptor family. Its predominant expression in white and brown adipose tissues, coupled with its critical functions in adipogenesis, lipid metabolism, and energy homeostasis, makes it a central player in metabolic health and disease [93]. GWAS studies have demonstrated that the *PPAR-γ* gene is among the candidate genes which are susceptible to T2D loci and SNPs in this nuclear receptor act in the control of the metabolism of lipid and glucose. The rs1801282 (C > G) SNP has been extensively studied by researchers and data from a recent meta-analysis and systematic review showed that the variant G allele was associated with a decreased risk of T2D in different genetic models. Considering that T2D is a polygenic disease, a cross-sectional prospective study conducted in Saudi Arabia demonstrated that the combination of the CC genotype for rs1801282, AA for *FTO* rs9939609, and CC for *MC4R* rs2229616 increased the risk of the disease [94]. In addition, a study involving 300 pregnant women verified that carriers of CG and GG alleles presented a lower risk for GDM [95].

The effect of two diets was evaluated in a study involving postmenopausal women with central obesity, all of whom were genotyped for rs1801282. The dietary intervention period was 16 weeks and included two groups: one following the MedDiet and the other following a Central European diet (CED), which was moderated in the intake of carbohydrates. After the intervention period, the carriers of the G allele in the CED group showed a significant reduction in weight, lean mass, and HDL-c, contributing to an improvement in IR. In the MedDiet group, G allele carriers also had a significant reduction in abdominal fat compared to those with the C allele [96].

A study involving 1465 overweight/obese Spanish individuals undergoing a 2-year behavioral weight-loss program based on the MedDiet investigated the interaction between the *PPARG* rs1801282 SNP and dietary fat intake. The program encompassed dietary guidance, physical activity recommendations, and behavioral strategies. In the baseline, the CG/GG genotypes presented lower insulin concentrations and HOMA-IR compared to the CC genotype. After the intervention period, the carriers of the minor G allele had an increased percentage of weight loss and body fat, especially in those with high MUFA [97].

A study conducted with T2D patients from Turkey observed a positive association between the total fat intake and waist circumference in individuals with the CC genotype. For the CG/GG genotypes, no association was observed [98]. A randomized crossover clinical trial investigated the effect of the interaction between the *PPARG* rs1801282 SNP and dietary fat intake on serum lipids in 34 non-diabetic men selected from the METSIM study. The participants followed both a high-SAFA diet and a high-PUFA diet for 8 weeks each. In this case, individuals with the CC genotype, during the PUFA diet, exhibited significantly increased HDL-c and APOA-1 concentrations [99].

A randomized clinical trial with obese patients (BMI ≥ 40 kg/m^2^) investigated the influence of the *PPARG* rs1801282 SNP on body composition changes after a 12-week nutritional intervention. The participants were divided into groups that received either extra virgin olive oil supplementation (50 mL/day), a traditional Brazilian diet, or a traditional Brazilian diet with extra virgin olive oil supplementation. The participants were also divided into groups according to CC or CG/GG genotype. The intervention with a traditional Brazilian diet and supplementation with extra virgin olive oil promoted weight loss and improved the body composition in patients with severe obesity, regardless of the rs1801282 genotype [100]. Another study conducted with T2D patients demonstrated that docosahexaenoic acid-rich fish oil supplementation (2 g/day) reduced the body weight, waist circumference, fasting glucose, and HOMA-IR of individuals independently of their rs1801282 genotype [101].

#### 3.2.8. Adiponectin

Adiponectin (ADIPOQ), an adipokine abundantly expressed in white adipocytes, is related to IR, obesity, atherosclerosis, and T2D [102,103,104]. The *ADIPOQ* gene is in chromosome 3q27, is composed of three exons and two introns, and has been extensively studied for its associations with metabolic disorders. Several studies have reported that SNPs, including a silent T to G substitution (rs2241766; T > G) in exon 2 and a G to T substitution (rs1501299; G > T) on intron 2, are associated with a lower blood ADIPOQ concentration, IR, and risk of T2D [104]. The plasma ADIPOQ concentration is closely correlated with IR and is typically decreased in T2D patients [105]. This relationship can be explained by the fact that ADIPOQ enhances the insulin action on target tissues, thereby improving insulin sensitivity [106]. It has been observed that subjects who present the G allele of the rs2241766 polymorphism have lower plasma concentrations of ADIPOQ [107].

Additionally, for the SNP rs266729, the G allele has been identified as a risk factor for T2D. Individuals carriers of the GG and CG genotypes have a higher risk of developing T2D compared to those with the C allele [105]. Furthermore, the TT and TG genotypes for SNP rs2241766 have been significantly associated with poor glycemic control, highlighting its potential impact on diabetes management [106].

A significant dose–response interaction between the SNP rs1501299 (G > T), carbohydrate intake, and HbA1C was found in a study with the Korean population. It has been suggested that the influence of the SNP rs1501299 on HbA1C may not be due to differences in IR. The genetic background of glucose homeostasis is likely polygenic, implying that the rs1501299 polymorphism might be in linkage disequilibrium with another polymorphism that has functional relevance and which affects glucose metabolism, since rs1501299 is in an intron away from the consensus splice site [108].

Another study suggests that the T allele of rs1501299 SNP could be a predictor of a lack of response in HOMA-IR insulin, fasting glucose, and LDL-c secondary to a Mediterranean hypocaloric diet in obese subjects [109]. Furthermore, it was observed that the genetic variant rs2241766 (G > A) increased the serum ADIPOQ concentration after the intake of a high-monounsaturated diet, whereas in A allele carriers, the serum ADIPOQ concentration decreased [110]. This differential response was not detected with a low-fat hypocaloric diet [109]. In conclusion, ADIPOQ plays a crucial role in IR, obesity, atherosclerosis, and T2D, with specific SNPs being closely linked to these conditions. Genetic variations such as rs266729 and rs2241766 further illustrate the complexity of gene–diet interactions and their impact on metabolic health.

**Figure 2:**
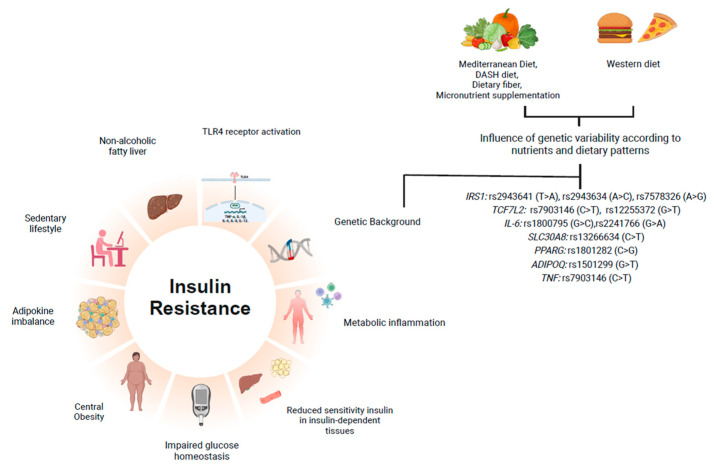
Influence of nutrients and dietary patterns on single-nucleotide polymorphisms related to lifestyle and metabolic impairments involved in insulin resistance development.

Table 1 summarizes the main effects of nutrients and dietary patterns on SNPs reported by the studies reviewed in this study, while Figure 2 provides a visual representation of how these nutrients and dietary patterns influence genetic polymorphisms related to metabolic impairments involved in IR, offering a comprehensive overview of the interactions discussed in this section.

### 3.3. Effects of Nutritional Interventions on Polygenic Risk Scores Affecting IR Biomarkers

The polygenic risk score (PRS) or genetic risk score (GRS) is a robust tool used to accurately estimate an individual’s genetic risk to various traits and diseases. The calculation of this score is obtained by a mathematical aggregate that considers several SNPs from GWAS studies to estimate the likelihood of risk for specific outcomes. Several models of PRS can differ in the number of SNPs considered, the type of statistical model used to assess the associated risks, and the punctuation capacity to generalize this risk for all the studied population [111,112]. Despite their potential, there are few studies investigating the effects of dietary interventions on PRS and GRS and their impact on IR biomarkers, highlighting a necessity for more research to understand gene–diet interactions.

A PRS was constructed from SNPs that demonstrated associations with glucose and insulin metabolism, as well as obesity, in multiple ethnic groups in a study involving 200 Brazilian young adults. The evaluation of the dietary intake of macronutrients was performed using 3-day food records. The individuals within the high-fat intake category and with ≥5 risk alleles presented a significantly higher fasting insulin, insulin–glucose ratio, HOMA-β, and HOMA-IR than those with <5 risk alleles [113]. In another study, a GRS calculated from 15 SNPs associated with metabolic traits did not show an influence on dietary protein intake or the glucose concentration, insulin level, and HbA1C in a study with 110 women from Indonesia [114].

A study conducted with 106 healthy women from Minangkabau Indonesia Study, constructed a PRS with 23 SNPs associated with metabolic traits and verified their association with dietary intake. Participants with a low MUFA intake (<7 g/day) and a higher GRS (>13 risk allele) presented significantly higher HbA1c levels compared to those with a low MUFA intake and GRS (≤13 risk allele) [115].

An interventional study that involved 138 participants with a BMI ranging from 25 to 40 kg/m^2^ who received an omega-3 supplementation consisting of 5 g/day of fish oil (1.9–2.2 g of eicosapentaenoic acid (EPA) and 1.1 g of docosahexaenoic acid (DHA)) for six weeks. A PRS was developed based on eight SNPs that demonstrated a significant association with changes in the HOMA-IR following n-3 PUFA supplementation. The results revealed that participants with a higher PRS showed a more pronounced increase in HOMA-IR after omega-3 supplementation, suggesting a decline in insulin sensitivity. These findings highlight the relevant role of the genetic background in the interindividual variability observed in the insulin sensitivity response to n-3 PUFA supplementation. Identifying individuals at risk of reduced insulin sensitivity through the use of genetic-based precision nutrition approaches may help to tailor more effective dietary interventions [116].

The interaction between obesity-related GRS, derived from the HELENA (Healthy Lifestyle in Europe by Nutrition in Adolescence) study cohort, and MedDiet adherence was comprehensively evaluated in a cohort of 605 European adolescents. This study revealed a significant sex-specific effect, particularly pronounced among female participants. Specifically, in female subjects with an elevated obesity-GRS, adherence to the MedDiet was associated with marked improvements in insulin resistance, as quantified by the HOMA-IR index [117].

Watanabe and collaborators (2018) investigated an association between postprandial plasma glucose levels and a GRS constructed using five SNPs related to T2D risk in young non-diabetic Japanese individuals with risk alleles for T2D who consumed 150 g of yogurt for 4 weeks. The group with a higher GRS presented an improvement in their postprandial plasma glucose level, insulin level, and HOMA-IR after the intervention with yogurt compared to those with low GRS [118].

In the context of sugar-sweetened beverages (SSBs), López-Portillo and collaborators (2021) studied the association between SSBs and the genetic risk for T2D and its effects on fasting glucose. A total of 2828 individual residents in Chile were evaluated regarding their SSB consumption and classified into categories based on their frequency of consumption (1 portion = 350 mL). The GRS was calculated using 16 SNPs associated with T2D. The authors verified that the consumption of SSBs was associated with an increased glucose concentration, especially in individuals with a high GRS [119].

The PRS can be used to identify individuals at high risk for IR and related metabolic disorders, allowing for early intervention and targeted prevention strategies. This tool holds great promise for personalized nutrition, but the clinical applicability of PRS still faces some challenges. There is a lack of a standardized PRS for IR, making it difficult to compare results across studies. Future studies should focus on developing a standardized PRS, validating their accuracy in diverse populations, and conducting implementation studies to assess their impact on clinical outcomes.

Table 2 provides the details of the SNPs that comprise the PRSs/GRSs in the aforementioned studies, highlighting the impact of nutrients and dietary interventions.

## 4. Conclusions

This review highlighted the critical effect of the interplay between genetic and dietary factors on the modulation of IR biomarkers. The identification of SNPs associated with IR and the construction of PRSs has advanced our understanding of genetic predisposition to this condition. However, it also reveals that individuals may exhibit different responses to nutritional interventions based on their genotype or the degree of a PRS, which significantly affect the prognosis of IR. Future research should continue to explore these aspects of gene–diet interactions, prioritizing large-scale, multi-ethnic studies to validate these findings across diverse populations and longitudinal studies to assess the long-term effects of personalized dietary interventions. These findings have the potential to contribute to dietary guidelines and enable healthcare professionals to tailor interventions and improve health outcomes. Studies investigating the underlying mechanisms of these interactions to elucidate other potential molecular pathways related to these interactions and develop more precise and effective approaches to managing IR and its associated complications are also relevant.

## Figures and Tables

**Table 1:** SNPs associated with insulin resistance and metabolic inflammation and their influence on different nutrients and dietary interventions.

Gene	SNPs	Nutrients and/or Nutritional Intervention	Main Findings
*TCF7L2*	rs7903146 (C > T) and rs12255372 (G > T)	Mediterranean Diet	(1) T allele for both SNPs: individuals with high adherence to the Mediterranean diet presented ↓ weight, BMI, WC [56], and lower risk for GDM [57]; (2) individuals with low adherence had higher fasting glucose [55].
	rs7903146 (C > T)	Quantitative evaluation of dairy products (>200 mL/day), processed sweets (>30 g/day), and refined grains (≥5 portions/week)	T allele: individuals with higher consumption of desserts and milk had ↑ risk for T2D [52].
		Evaluation of habitual intake of SAFA (low intake ≤ 12% TEV and high intake ≥ 15%TEV)	T allele: ↑ intake of SAFA associated with impaired insulin sensitivity [53].
		Evaluation of intake of whole grains (>50 g/day vs. <30 g/day)	GG genotype: 14% less risk of developing T2D [54].
		DASH diet or legume-based DASH diet	No effects of the SNP on inflammatory biomarkers or oxidative stress indicator [58].
*SLC30A8* rs13266634 (C > T)		Zinc intake	TT genotype: zinc supplement users had a more pronounced reduction in T2D risk [65].
		Supplementation of oral zinc acetate	(1) CT/TT genotypes: ↑ fasting insulin. (2) No differences between genotypes for serum zinc concentration [66].
		MMTT test with high lipids content (60% lipids; 25% carbohydrates; 15% proteins)	(1) CC genotype: ↓ NEFA in men. (2) TT genotype carriers: ↑ insulin basal levels in women and attenuated lipid response with no gender-specific effect [67].
*IRS1*	rs2943641 (T > A)	Macronutrients intake	T allele: ↓ T2D risk in lower tertiles of carbohydrate intake for women and in the lower tertiles of fat intake for men [71].
	rs2943634 (A > C)	Counseling program: reduction in refined carbohydrates (<45% TEV), emphasis on dietary fiber (>25 g/day)	A allele: associated with ↓ carbohydrate intake [72].
	rs2943641 (T > A) and rs7578326 (A > G)	Dietary analysis of SAFA: carbohydrates (Group 1: Ratio ≤0.24 and Group 2: Ratio >0.24) and MUFA intake	(1) haplotype G-T: ↓ risk of IR and MetS with low dietary SAFA-to-carbohydrate ratio. (2) G allele: ↓ MetS risk when dietary MUFA was lower than median intake [27].
*TNF*	rs7903146 (C > T)	Mediterranean Diet	GG genotype: improvement of TG and plasma hsCRP after 1 year of intervention [77].
		Fatty acids intake evaluation	A allele: ↑ inflammation— ↑ plasma stearic acid and SAFA [74].
*TLR4*	rs4986790 (Asp299Gly) and rs5030718 (G > A)	Dietary fat intake evaluation	(1) Gly allele: ↑ insulin, HOMA-IR, and HOMA-β. (2) G allele: SFA intake -> inversely associated with HDL-c among GG individuals and a positive relationship for GA [85]
*IL6*	rs1800795 (G > C)	3 intervention groups: (a) lower fat and higher carbohydrate; (b) lower carbohydrate and higher fat; or (c) a walnut-rich diet, with higher fat and lower carbohydrate.	CC genotype: ↓ IL-6 levels before adjustment for BMI [92].
*PPARG*	rs1801282 (C > G	Mediterranean Diet or Centro-European diet	(1) Central European diet—G allele: ↓ weight, lean mass, and HDL-c. (2) Mediterranean Diet—G allele: ↓ abdominal fat [96].
		2-year behavioral weight-loss program based on Mediterranean Diet	(1) CG/GG genotype: baseline—↓ insulin and HOMA-IR; (2) G allele: after intervention—↑ % of weight loss and body fat, especially in those with high MUFA intake [97].
		High SAFA content or high PUFA intake	(1) CC genotype: a positive association between total fat intake and WC [98]; (2) PUFA diet—↑ HDL-c and APOA [99].
		3 interventional groups: extra virgin olive oil supplementation (50 mL/day), a traditional Brazilian diet (TBD), or a TBD with extra virgin olive oil supplementation.	TBD with extra virgin olive oil supplementation: weight loss and improvement body composition independently of genotype [100].
			↓ body weight, fasting glucose, and HOMA-IR independent of genotype [101].
		Daily DHA-rich fish oil supplementation	
*ADIPOQ*	rs1501299 (G > T)	Carbohydrate intake evaluation	T allele carriers influence the degree to which fasting glucose, HbA_1_C e HDL-c are affected by the amount of carbohydrate intake [108].
	rs2241766 (G > A)	High-monounsaturated diet	A allele: ↓ serum adiponectin [110].

SNP: single-nucleotide polymorphism; *TCF7L2*: transcription factor 7-like 2; *IRS1*: insulin receptor substrate 1; BMI: body mass index; WC: waist circumference; DASH: Dietary Approaches to Stop Hypertension; GDM: gestational diabetes melitus; SAFA: saturated fatty acid; MUFAs: monounsaturated fatty acids; NEFA: non-esterified fatty acids; *TNF*: tumor necrosis factor-alpha; *IL6*: interleukine 6; TEV: total energy value; MDA: malondialdehyde; T2D: type 2 diabetes; MMTT: mixed meal tolerance test; *TLR4*: toll-like receptor 4; MetS: metabolic syndrome; IR: insulin resistance; hs-CRP: high sensitive C-reactive protein; TG: triglycerides; HOMA-IR: homeostasis model assessment-estimated insulin resistance; HOMA-β: homeostasis model assessment-beta cell; *PPARG*: peroxisome proliferator-activated receptor gamma; PUFAs: polyunsaturated fatty acids; APOA-1: apolipoprotein A1; DHA: docosahexaenoic acid; *ADIPOQ*: adiponectin.

**Table 2:** Influence of different PRSs/GRSs on nutrients and dietary patterns.

PRS/GRS Composition	Nutrients and/or Nutritional Intervention	Nutrients and/or Nutritional Intervention
10 SNPs associated with glucose and insulin metabolism, and obesity (rs12255372, rs7903146, rs17782313, rs8050136, rs10163409, rs2237892, rs2237895, rs10811661, rs5030952, rs1801282)	Macronutrients intake	Individuals within the high-fat intake category and with ≥5 risk alleles: ↑ fasting insulin, insulin-glucose ratio, HOMA-β, and HOMA-IR than those with < 5 risk allele [113].
16 SNPs associated with metabolic traits (rs3792267, rs5030952, rs9939609, rs10163409, rs8050136, rs17782313, rs2229616, rs12255372, rs7903146, rs2237895, rs2237892, rs10811661, rs1801282, s266729, rs17846866)	Protein intake evaluation	No influence of PRS on dietary protein intake and glucose concentration, insulin, and HbA1C [114].
23 SNPs associated with metabolic traits (rs1801133, rs7903146, rs12255372, rs8050136, rs9939609, rs17782313, rs1801282, rs5219, rs2237895, rs2237892, rs10741567, rs12794714, rs12785878, rs6013897, rs2282679, rs6680429, rs266729, rs10811661, rs1801725, rs5030952, rs3742801, rs2270655, rs778805)	MUFA intake evaluation	Women with ↓ MUFA intake (<7.0 g/day) and a higher PRS (>13 risk allele) had elevated HbA1C [115]
8 SNPs associated with changes in the HOMA-IR after n-3 PUFA supplementation (rs72723587, rs77850702, rs72703546, rs17174795, rs12437986, rs35621498, rs55842940, rs6001872)	Omega-3 PUFA supplementation (5 g/day of fish oil)	↑ PRS increased HOMA-IR after dietary intervention [116]
21 SNPS associated with obesity (rs2010899, rs4135275, rs4912905, rs9355296, rs1524107, rs2183013, rs1019731, rs1568400, rs4246444, rs7701443, rs13182800, rs3211867, rs2515362, rs1800497, rs9939609, rs4783961, rs8068149, rs7502966, rs1044250, rs17373080, rs2143511).	Mediterranean Diet	Women with adherence to the intervention diet and ↑ PRS: improvement of IR [117]
5 SNPs associated with T2D risk (rs2237892, rs2206734, rs2383208, rs6780569, rs1470579)	Intake of 150 g of yogurt	↑ PRS group: improvement of postprandial plasma glucose, insulin, and HOMA-IR after the intervention [118]
16 SNPs associated with T2D (rs3923113, rs1801282, rs11717195, rs4402960, rs6878122, rs7756992, rs849135, rs516946, rs17791513, rs2796441, rs11257655, rs7903146, rs10830963, rs7403531, rs7202877, rs5945326, rs188827514)	Sugar-sweetened beverages (SSB) intake evaluation	SSB intake: increased glucose levels in individuals with ↑ PRS [119]

SNP: single-nucleotide polymorphism; PRS: polygenic risk score; GRS: genetic risk score; HOMA-IR: homeostasis model assessment-estimated insulin resistance; HOMA-β: homeostasis model assessment-beta cell; PUFAs: polyunsaturated fatty acids; MUFAs: monounsaturated fatty acids; IR: insulin resistance; T2D: type 2 diabetes.

## Data Availability

No new data were created or analyzed in this study. Data sharing is not applicable to this article.

## Abbreviations

ADIPOQ:	Adiponectin
APOA-1:	Apolipoprotein A-1
BMI:	Body Mass Index
DASH:	Dietary Approaches to Stop Hypertension
DNL:	De Novo Lipogenesis
FTO:	Fat Mass and Obesity Associated
GWAS:	Genome-Wide Association Studies
HbA1c:	Glycated Hemoglobin
HDL:	High-Density Lipoprotein
HOMA-IR:	Homeostasis Model Assessment of Insulin Resistance
IL-6:	Interleukin-6
IRS:	Insulin Receptor Substrate
IR:	Insulin Resistance
JNK:	c-Jun N-terminal Kinase
MedDiet:	Mediterranean Diet
MC4R:	Melanocortin-4 Receptor
MUFA:	Monounsaturated Fatty Acids
NF-κB:	Nuclear Factor Kappa B
PUFA:	Polyunsaturated Fatty Acids
PRS:	Polygenic Risk Score
SNP:	Single-Nucleotide Polymorphism
SAFA:	Saturated Fatty Acids
TCF7L2:	Transcription Factor 7-Like 2
TLR:	Toll-Like Receptor
TNF-α:	Tumor Necrosis Factor-Alpha
T2DM:	Type 2 Diabetes
TyG:	Triglyceride-Glucose Index
GRS:	Genetic Risk Score
PRS:	Polygenic Risk Score
